# Sex-Specific Associations of Total Bilirubin, ALBI, and PALBI with Lung Cancer Risk: Interactions with Smoking and Alcohol

**DOI:** 10.3390/healthcare13111321

**Published:** 2025-06-02

**Authors:** Jong Won Shin, Nguyen Thien Minh, Sun Ha Jee

**Affiliations:** 1 Department of Laboratory Medicine, Asan Medical Center, University of Ulsan College of Medicine, Seoul 05505, Republic of Korea; jongwon_shin@amc.seoul.kr; 2 Department of Epidemiology and Health Promotion, Institute for Health Promotion, School of Public Health, Yonsei University, Seoul 03772, Republic of Korea; 3 Department of Epidemiology, Faculty of Public Health, University of Medicine and Pharmacy, Ho Chi Minh City 17000, Vietnam; nguyenminh2301@gmail.com

**Keywords:** lung neoplasms, bilirubin, smoking, alcohol drinking, sex factors

## Abstract

**Background:** Bilirubin is a potent endogenous antioxidant that plays a key role in regulating oxidative stress and inflammation, both closely linked to lung carcinogenesis. This study reinterprets the ALBI (Albumin–Bilirubin) and PALBI (Platelet–Albumin–Bilirubin) indices as composite markers of antioxidant and inflammatory status and evaluates their associations with lung cancer risk by sex, including stratified analyses by major lifestyle factors such as smoking and alcohol use. **Methods:** This study utilized data from the Korean Cancer Prevention Study-II (KCPS-II) cohort, which included 133,630 participants. During a mean follow-up of 13.5 years, 721 incident lung cancer cases were identified. Serum bilirubin and the ALBI and PALBI indices were analyzed by sex, and quartile-based and trend analyses were conducted. Stratified analyses by smoking and alcohol status (never, former, current, ever) and intensity were performed to assess potential effect modification. Cox proportional hazards regression models were used to estimate HRs and 95% CIs. **Results**: A 1SD increase in total bilirubin and ALBI was inversely associated with lung cancer risk in men (HR: 0.83, 95% CI: 0.75–0.91; HR: 0.86, 95% CI: 0.79–0.94, respectively), whereas PALBI showed a positive association (HR: 1.17, 95% CI: 1.07–1.28). In contrast, in women, total bilirubin and ALBI showed positive associations (HR: 1.19, 95% CI: 1.00–1.40; HR: 1.19, 95% CI: 1.02–1.40, respectively), while PALBI was inversely associated (HR: 0.82, 95% CI: 0.69–0.97). These associations were significant among men who were smokers (former, current, ever) and men who drank alcohol (current, ever), whereas in women, significance was observed only among never drinkers. Stronger interactions were observed in men who were heavy smokers and low-to-moderate drinkers. **Conclusions:** Bilirubin and the ALBI and PALBI indices exhibit sex-specific and contrasting associations with lung cancer risk, highlighting the need to consider sex-based physiological differences in cancer risk assessment.

## 1. Introduction

Lung cancer remains one of the leading causes of cancer-related mortality worldwide [1,2]. Numerous traditional blood-based biomarkers have been investigated to predict its development and progression. Commonly studied markers include carcinoembryonic antigen (CEA), C-reactive protein (CRP), lactate dehydrogenase (LDH), neutrophil-to-lymphocyte ratio (NLR), lymphocyte-to-monocyte ratio (LMR), platelet-to-lymphocyte ratio (PLR), and the advanced lung cancer inflammation index (ALI), all of which have been widely examined as prognostic or risk assessment indicators for lung cancer [3,4,5,6,7]. The most critical risk factor for lung cancer is smoking, which acts as a potent inducer of oxidative stress. Smoking disrupts redox homeostasis and triggers chronic inflammatory responses, thereby contributing to the initiation and progression of lung cancer [8]. Biomarkers associated with these mechanisms provide essential insight into the pathophysiological processes of lung cancer and enable more precise individual risk stratification.

Bilirubin, a byproduct of heme catabolism, functions as a powerful endogenous antioxidant. It scavenges reactive oxygen species (ROS), alleviates oxidative stress, and helps maintain redox balance by regulating the NADH/NAD⁺ ratio, thereby suppressing ROS production at the source. Through these mechanisms, bilirubin inhibits ROS-induced DNA damage and inflammation, reducing the formation of a tumor-promoting microenvironment [9,10,11,12,13,14,15]. Recent epidemiological and Mendelian randomization (MR) studies have demonstrated a causal inverse association between genetically elevated bilirubin levels and lung cancer risk [16]. Moreover, bilirubin has been implicated in the risk modulation of several other cancers, including colorectal, breast, cervical cancers, and melanoma [17,18,19,20].

Albumin also acts as a non-enzymatic antioxidant by binding with metal ions and suppressing ROS generation through the inhibition of Fenton-type reactions. In addition, albumin enhances the bioavailability of bilirubin through complex formation, and the two molecules work synergistically to neutralize ROS and inhibit inflammatory signaling [9,10,11,12,13,14,15,21]. Persistent ROS accumulation activates the NF-κB pathway, leading to the expression of pro-inflammatory cytokines such as IL-6 and TNF-α, thereby promoting chronic inflammation [21,22,23]. Bilirubin and albumin both attenuate this pathway, while albumin further supports intracellular redox balance by maintaining the glutathione system, thus playing a crucial role in preventing early carcinogenic changes [21].

Platelets, traditionally known for their role in hemostasis, also contribute significantly to the tumor microenvironment. Platelet-activating factor (PAF), secreted by platelets, enhances tumor invasiveness and facilitates interactions between platelets and tumor cells, promoting metastasis [24,25,26,27]. Activated platelets are closely linked to ROS generation, further amplifying inflammatory responses and cancer progression.

Given these physiological characteristics, the present study aimed to evaluate not only the independent antioxidant role of bilirubin but also the utility of composite indices that incorporate albumin and platelet counts. The Albumin–Bilirubin (ALBI) index reflects hepatic functional reserve based on serum albumin and bilirubin levels, while the Platelet–Albumin–Bilirubin (PALBI) index adds platelet count to account for portal hypertension and inflammation-related factors [28,29]. Although originally developed to assess prognosis in hepatocellular carcinoma (HCC), ALBI and PALBI may serve as integrated biomarkers of antioxidant and inflammatory regulation due to the physiological roles of their components. Recent studies suggest that these indices may also have prognostic value in other solid tumors, including lung, breast, and colorectal cancers [30,31,32,33]. However, most existing studies have focused on patient populations, often using cross-sectional or retrospective designs that are susceptible to confounding by treatment status, cancer stage, and selection bias. Furthermore, many of these studies have been conducted primarily in male cohorts, limiting sex-specific interpretation. Given the long latency and progressive nature of lung cancer, large-scale prospective cohort studies are essential to elucidate its risk factors and underlying mechanisms.

In this context, we utilized data from the Korean Cancer Prevention Study-II (KCPS-II), a large-scale prospective cohort of the general population, to evaluate the associations between lung cancer risk and bilirubin as an independent antioxidant marker, along with the extended functions of the ALBI and PALBI indices. Considering smoking and alcohol consumption as major behavioral risk factors for lung cancer, we further conducted stratified analyses by smoking status (never, former, current, and ever smokers) and smoking intensity (cigarettes per day), as well as alcohol consumption status (never, former, current, and ever drinkers) and drinking intensity. These subgroup analyses aimed to assess whether the ALBI and PALBI indices could function not only as predictors of lung cancer risk but also as sensitive physiological indicators of antioxidant and inflammatory responses according to individual exposure levels. Importantly, the associations between these indices and alcohol-related parameters (drinking status and intensity) in the context of lung cancer have not been explored in previous studies, highlighting the novelty of our approach. Thus, our findings provide new evidence that the ALBI and PALBI indices may serve as integrative markers for elucidating the pathophysiological mechanisms of alcohol-related lung carcinogenesis, extending their applicability beyond traditional prognostic use.

## 2. Methods

### 2.1. Study Population

This study utilized data from the Korean Cancer Prevention Study-II (KCPS-II), which included 153,971 adults aged 20 years or older who visited 18 health screening centers across South Korea between 2004 and 2013. All participants provided written informed consent for the use of their health screening and questionnaire data for research purposes [34]. Individuals who had been diagnosed with cancer or were cancer survivors prior to cohort enrollment were excluded from this study. Additionally, those with missing data on smoking and alcohol consumption were excluded, resulting in a preliminary sample of 143,282 participants. After further excluding participants with missing measurements of bilirubin, albumin, or platelet count, a total of 133,596 participants were included in the final analysis. The characteristics of the study population are summarized in Table 1.

Of the 133,596 participants, 83,371 were men and 50,225 were women. The mean age of the total population was 40.9 years (SD 10.0), with men having a mean age of 41.6 years (SD 9.5) and women 39.7 years (SD 10.7). The analyzed variables included body mass index (BMI), serum total bilirubin, albumin, platelet count, smoking status, and alcohol consumption. Smoking status was classified as never-smokers, former smokers, current smokers, and ever smokers (including both former and current smokers). Alcohol consumption was categorized as never-drinkers, former drinkers, current drinkers, and ever drinkers (including both former and current drinkers). Information on smoking and alcohol consumption was collected through baseline self-reported questionnaires, based on the cohort profile of the KCPS-II Biobank [34]. This study was approved by the Institutional Review Board (IRB) of Severance Hospital, Yonsei University Health System (Approval No. 4–2011–0277).

### 2.2. Cancer Case Ascertainment

The occurrence of cancer among study participants was verified annually with near-complete accuracy by linking to the Korea National Cancer Center (NCC) cancer registry using unique resident registration numbers [34]. In South Korea, under the Cancer Control Act, all hospitals are legally required to report cancer diagnoses to the NCC, ensuring comprehensive cancer registration nationwide. There were no dropouts due to withdrawal of informed consent after enrollment in the cohort. Cancer cases were classified according to the 10th revision of the International Classification of Diseases (ICD-10). The mean follow-up duration for the entire cohort was 13.5 years. Specifically for lung cancer (ICD-10: C34), the median follow-up period was 14.0 years (interquartile range: 13.4–14.6 years), and a total of 721 incident lung cancer cases were identified.

### 2.3. Statistical Analysis

Descriptive statistics were used to summarize the baseline characteristics of the study population. Total bilirubin and the ALBI and PALBI indices were each categorized into quartiles to evaluate their associations with lung cancer incidence, and trend tests were performed to assess dose–response relationships across quartiles. Additionally, the associations between 1-standard-deviation (1 SD) increases in total bilirubin, ALBI, and PALBI levels and lung cancer risk were assessed. Cox proportional hazards regression models were used to estimate the associations between these biomarkers and the incidence of lung cancer, and the results were presented as hazard ratios (HRs) with 95% confidence intervals (CIs). All models were adjusted for potential confounders, including age, smoking status, alcohol consumption, body mass index (BMI), serum glutamate oxaloacetate transaminase (GOT), and gamma-glutamyl transferase (GGT). Age and BMI were included as key confounding variables because they are well-established demographic and metabolic risk factors for lung cancer. In particular, BMI has been shown to have an inverse dose–response association with lung cancer risk in a recent meta-analysis [35] and was therefore considered essential for adjustment in this study. GOT (AST) and GGT were included not only as indicators of liver function but also because of their relevance to oxidative stress and antioxidant regulation. GGT plays a central role in glutathione (GSH) metabolism, and GSH is a potent endogenous antioxidant involved in tumor progression and resistance to anticancer therapy [36]. Given that both bilirubin and albumin contribute to the body’s antioxidant defense system, adjusting for GOT and GGT allowed us to control for the potential confounding effects of hepatic function and redox status on the associations between bilirubin-related indices and lung cancer risk. Person-years of follow-up were calculated for each participant, and total bilirubin was analyzed both as a continuous variable and by quartiles as an independent antioxidant biomarker in the Cox regression models. The ALBI index was calculated using the following formula: ALBI = (log_10_ bilirubin [µmol/L] × 0.66) + (albumin [g/L] × −0.085) [28]. Bilirubin values were converted from mg/dL to µmol/L for this calculation. The PALBI index was calculated using the following formula: PALBI = log_10_(platelet count × 10^9^/L) − 2.02 × albumin (g/dL) − 0.37 × log_10_(bilirubin [mg/dL]) [29]. To minimize potential bias due to cancer latency, sensitivity analyses were performed by excluding lung cancer cases diagnosed within the first three years of follow-up. All biochemical measurements used in the analysis were obtained from laboratories that adhered to both internal and external quality control procedures in accordance with the standards of the Korean Association of Laboratory Quality Control. The inter-laboratory correlation coefficients for these measurements ranged from 0.96 to 0.99, indicating a high level of accuracy and consistency across testing centers [34].

## 3. Results

**Figure 1:**
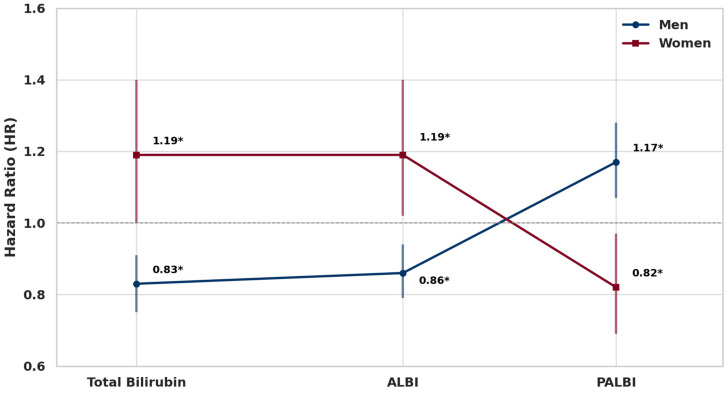
Sex-specific associations between a 1SD increase in total bilirubin, ALBI, and PALBI and lung cancer risk. Note: Error bars represent 95% confidence intervals. * *p* < 0.05.

Total bilirubin, ALBI, and PALBI showed statistically significant but opposite associations with lung cancer risk between men and women (Figure 1).

In men, a one-standard-deviation (1SD) increase in total bilirubin was associated with a 17% lower risk of lung cancer (HR: 0.83, 95% CI: 0.75–0.91, *p* = 0.0001), and ALBI showed a similar inverse association with a 14% risk reduction (HR: 0.86, 95% CI: 0.79–0.94, *p* = 0.0008). In contrast, PALBI was positively associated with lung cancer risk, showing a 17.2% increase per 1SD increment (HR: 1.17, 95% CI: 1.07–1.28, *p* = 0.0007). Among women, total bilirubin and ALBI were both positively associated with lung cancer risk, with 1SD increases corresponding to 19% higher risk (HR: 1.19, 95% CI: 1.00–1.40, *p* = 0.0473 for total bilirubin; HR: 1.19, 95% CI: 1.02–1.40, *p* = 0.0313 for ALBI). In contrast, PALBI was inversely associated with lung cancer risk, showing an 18% decrease (HR: 0.82, 95% CI: 0.69–0.97, *p* = 0.0207).

These sex-specific inverse patterns were consistently observed in quartile-based and trend analyses. In men, total bilirubin and ALBI levels in the Q3 and Q4 quartiles were linearly associated with lower lung cancer risk, while PALBI in Q3 and Q4 was linearly associated with increased risk. In contrast, in women, total bilirubin and ALBI in Q4 were linearly associated with increased lung cancer risk, whereas PALBI in Q4 showed a linear inverse association. All trend analyses were statistically significant (Table 2).

In analyses stratified by smoking status in men, total bilirubin and ALBI showed significant inverse associations with lung cancer risk across former smokers, current smokers, and ever smokers, while PALBI was positively associated in all smoking groups. No significant associations were observed in never smokers for any of the three markers (Table 3).

**Figure 2:**
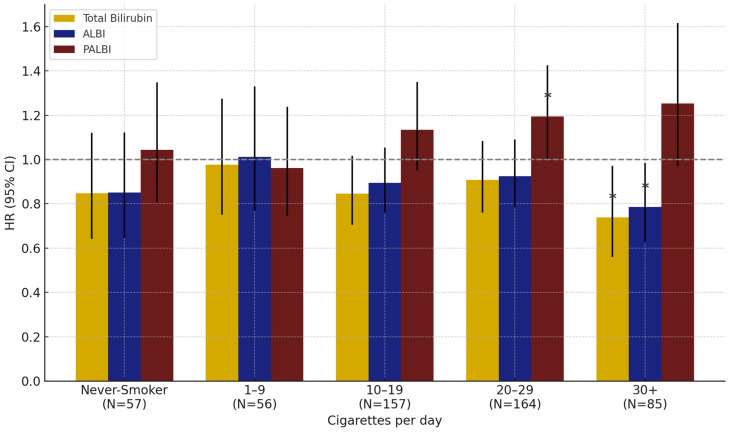
Effect of total bilirubin, ALBI, and PALBI on lung cancer risk by smoking intensity in men: results from interaction analyses. Note: Error bars represent 95% confidence intervals. * *p* < 0.05.

In analyses by smoking intensity, PALBI was positively associated with lung cancer risk in the group smoking 20–29 cigarettes per day (HR: 1.12, 95% CI: 1.00–1.43, *p* = 0.0482). Among those smoking ≥30 cigarettes/day, total bilirubin (HR: 0.74, 95% CI: 0.56–0.97, *p* = 0.0300) and ALBI (HR: 0.79, 95% CI: 0.63–0.80, *p* = 0.0045) were significantly associated with reduced risk. No significant associations were observed in other smoking intensity groups (Figure 2).

In men, stratified analyses by alcohol consumption status showed that total bilirubin, ALBI, and PALBI were significantly associated with lung cancer risk only among current and ever drinkers, but not among never-drinkers or former drinkers (Table 4).

In women, a significant positive association between total bilirubin and lung cancer risk was observed only among never-drinkers (Table 5).

**Figure 3:**
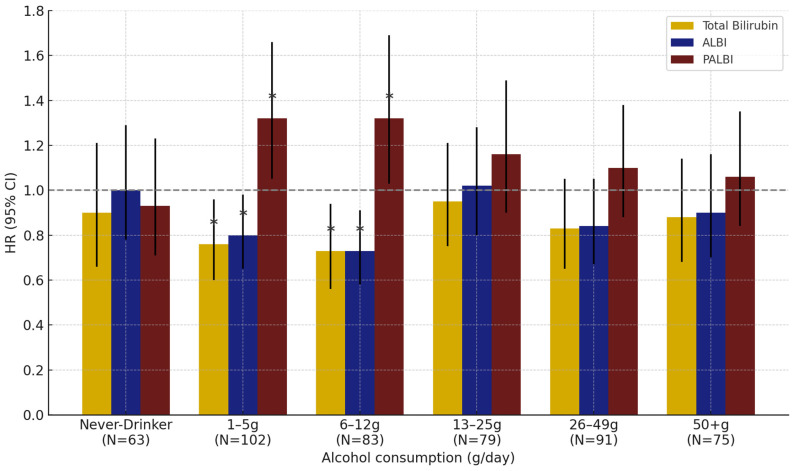
Effect of total bilirubin, ALBI, and PALBI on lung cancer risk by alcohol consumption in men: results from interaction analyses. Note: Error bars represent 95% confidence intervals. * *p* < 0.05.

In the subgroup analysis by alcohol intake level in men, all three markers showed statistically significant associations with lung cancer risk in the low (1–5 g/day) and moderate (6–12 g/day) alcohol consumption groups. No significant associations were found in other alcohol consumption groups, including never-drinkers (Figure 3).

## 4. Discussion

This study provides a novel perspective by demonstrating that serum bilirubin, the Albumin–Bilirubin (ALBI) index, and the Platelet–Albumin–Bilirubin (PALBI) index are differentially associated with lung cancer risk according to sex, based on a large-scale prospective cohort of healthy adults followed for approximately 14 years. These sex-based differences may reflect physiological distinctions in hormonal regulation, metabolic processing, and immune responses. Indeed, significant differences in serum bilirubin, albumin, and platelet levels between men and women have been previously reported [37,38,39]. Recent studies have also shown that sex hormones influence immune responses and PD-1 expression in lung cancer, with higher PD-1 expression frequently observed in female patients [40].

In this study, total bilirubin and the ALBI index were significantly inversely associated with lung cancer risk in men. These findings suggest that bilirubin may not only act as an antioxidant marker but also be involved in endocrine and tumor-suppressive physiological pathways. Recent research has shown that higher total bilirubin levels are significantly associated with elevated testosterone levels in men, whereas lower bilirubin concentrations are linked to an increased risk of testosterone deficiency [41,42]. This suggests that bilirubin may exert a protective endocrine effect by reducing oxidative stress in testicular tissue and supporting testosterone biosynthesis. In addition, albumin functions as the primary carrier of circulating testosterone, transporting it to target tissues via low-affinity binding mechanisms [43,44]. Therefore, reduced albumin levels may compromise the stability and biological activity of testosterone. Since the ALBI index integrates both bilirubin and albumin concentrations, the inverse association observed between ALBI and lung cancer risk in men may reflect a physiological state characterized by improved antioxidant capacity and hormonal balance. Epidemiological studies have further reported inverse associations between testosterone levels and lung cancer risk. Several large-scale cohort studies have consistently shown that men with higher total or free testosterone concentrations have a lower risk of developing lung cancer, supporting the hypothesis that testosterone may contribute to tumor suppression through anti-inflammatory, immunomodulatory, and cell-survival mechanisms [45,46]. Taken together, the observed inverse associations between total bilirubin and ALBI and lung cancer risk in men are biologically plausible given the interrelated pathways involving bilirubin–testosterone, albumin–testosterone, testosterone–lung cancer, and bilirubin–lung cancer. These findings support the notion that bilirubin and albumin may reduce lung cancer risk through mechanisms involving hormonal regulation, extending their role beyond that of simple antioxidant markers.

In contrast, among women, total bilirubin and ALBI were positively associated with lung cancer risk, while PALBI was inversely associated, showing an opposite pattern. These findings may reflect complex sex-specific differences in hormone metabolism, redox balance, and platelet physiology. Estrogen has been shown to induce the expression of CYP2A6 in the liver, promoting bilirubin metabolism [47], while bilirubin downregulates the OAT4 transporter, thereby reducing the cellular uptake of estrogen precursors and inhibiting estradiol synthesis [48]. Albumin has been reported to exhibit anti-estrogenic properties by inhibiting the proliferation of estrogen-receptor-positive cells [49], whereas estrogen has been shown to downregulate hepatic albumin mRNA expression and synthesis [50]. These bidirectional regulatory interactions among bilirubin, albumin, and estrogen constitute a complex physiological network that may influence lung cancer development in women. Estrogen is known to activate multiple tumor-promoting mechanisms in lung tissue via estrogen receptor β (ERβ), including enhanced cell proliferation, inhibition of apoptosis, elevated inflammatory responses, and promotion of metastasis. It is considered a major biological contributor to lung cancer risk in women [51,52,53]. In this context, the observed positive associations between total bilirubin and ALBI and lung cancer risk in women may reflect the combined effects of their interaction with estrogen-related metabolic pathways and hormone-regulated physiological mechanisms.

Meanwhile, the PALBI index, which incorporates platelet count along with albumin and bilirubin, reflects systemic inflammation and oxidative stress status. Platelets play a crucial role in the tumor microenvironment by promoting ROS generation and amplifying inflammatory signaling. In women, estrogen fluctuations are known to significantly influence platelet activation and antioxidant capacity [54,55,56,57]. Additionally, both platelets and their precursor megakaryocytes express estrogen receptor β (ERβ), and reductions in estrogen levels have been associated with decreased platelet antioxidant capacity and increased thrombotic potential [56,57]. These characteristics provide a physiological rationale for the observed inverse association between PALBI and lung cancer risk in women, in contrast to men. In men, PALBI was positively associated with lung cancer risk, suggesting that the increase in platelet count, combined with altered hormonal metabolism, may contribute to a tumor-promoting environment. As PALBI is composed of bilirubin, albumin, and platelets, differences in the metabolism and physiological functions of these components between sexes may underlie the divergent associations observed. In men, the PALBI index may reflect an inflammatory and pro-tumor state, whereas in women, it may indicate a hormonally modulated balance in redox and inflammatory regulation. These findings suggest that PALBI may function as a sensitive integrative marker that captures sex-specific tumor metabolic contexts and supports its potential use in evaluating the sex-dependent pathophysiology of lung cancer.

In this study, an analysis based on smoking status and smoking intensity among men demonstrated that total bilirubin and the ALBI and PALBI indices had significant associations with lung cancer risk. Notably, stronger associations were observed among smokers (former, current, and ever smokers) compared to never-smokers (Table 3, Figure 2). These findings are consistent with previous studies, suggesting that total bilirubin and the ALBI and PALBI indices may act as endogenous antioxidant markers that help protect lung tissue from oxidative stress induced by smoking. For example, a study based on a Korean cohort analyzing the combined effects of smoking and bilirubin reported that smokers with lower bilirubin levels had the highest risk of lung cancer, supporting the role of bilirubin as a protective factor mitigating oxidative damage [58]. In addition, an analysis using the UK Biobank found a significant inverse association between bilirubin and lung cancer risk only among current smokers, while no clear associations were observed in never-smokers or former smokers. This suggests that the antioxidant role of bilirubin may be more pronounced in environments with higher oxidative stress caused by smoking [16]. Similarly, findings from the Southern Community Cohort Study in the United States showed that lower pre-diagnostic levels of total bilirubin and albumin were associated with an increased risk of lung cancer, with this pattern being particularly evident among smokers. These results further emphasize the importance of antioxidant biomarkers in the context of smoking-related oxidative stress [59]. In particular, this study found that in the group of heavy smokers (≥20 cigarettes per day), total bilirubin and the ALBI and PALBI indices all showed significant associations with lung cancer risk, with these associations appearing more pronounced in this high-exposure group. This suggests that bilirubin and related indices may reflect more sensitive physiological responses to carcinogenic stress in environments of high oxidative burden.

In this study, an analysis based on alcohol consumption status and intake levels revealed that total bilirubin and the ALBI and PALBI indices were significantly associated with lung cancer risk among male current and ever drinkers, whereas in females, significant associations were observed in the never drinker group. These findings suggest that the physiological effects of alcohol on antioxidant and metabolic markers may differ by sex. Previous studies have failed to consistently demonstrate a direct association between alcohol consumption and lung cancer. Several large-scale cohort and meta-analytic studies have reported a tendency for increased lung cancer risk among heavy drinkers (≥30 g/day), but most failed to confirm a statistically significant linear association [60,61,62]. Moreover, the strong confounding effect of smoking has often hindered the independent evaluation of alcohol’s impact on lung cancer risk [60,61]. However, in the present study, after adjusting for smoking status as a confounder, significant associations between alcohol consumption and total bilirubin and the ALBI and PALBI indices were observed in both men and women. Notably, among men, these associations were more prominent in light-to-moderate drinkers than in heavy drinkers. This suggests that moderate alcohol intake may influence hepatic function and oxidative balance in a way that is sensitively reflected by bilirubin, as well as by composite indices incorporating albumin and platelet levels, such as ALBI and PALBI. These indices may therefore be useful in capturing physiological responses related to alcohol intake.

### 4.1. Conclusions

This large-scale prospective cohort study confirmed that total bilirubin, the Albumin–Bilirubin (ALBI) index, and the Platelet–Albumin–Bilirubin (PALBI) index are significantly associated with lung cancer risk, with the direction of these associations differing markedly between men and women. Notably, these biomarkers showed stronger associations among male smokers compared to never-smokers, and the interaction between these indices and lung cancer risk became more pronounced among heavy smokers. Similarly, consistent patterns were observed in male drinkers, with the strongest associations appearing among light-to-moderate drinkers, rather than heavy drinkers. In contrast, in women, significant associations were identified only among never drinkers, rather than current or former drinkers. These findings suggest that total bilirubin, ALBI, and PALBI may serve as valuable biomarkers for investigating the sex-specific and lifestyle-related pathophysiological mechanisms underlying lung cancer.

### 4.2. Limitations

Several limitations should be considered when interpreting the findings of this study. First, due to the observational nature of this study, causal relationships cannot be established, and further experimental research is needed to clarify the underlying biological mechanisms. Second, smoking data were self-reported, which may have introduced recall or reporting bias. Third, as the study population consisted of Korean individuals, the generalizability of the findings to other populations with different genetic and environmental backgrounds may be limited. Fourth, we were unable to account for several well-known environmental and occupational risk factors for lung cancer, such as asbestos, radon, diesel exhaust, air pollution (PM2.5), and chronic respiratory diseases. Fifth, although sex-specific differences were observed, we did not consider menopausal status or the use of hormone replacement therapy (HRT) among women, which may influence bilirubin metabolism and inflammatory responses. Sixth, the ALBI and PALBI indices may be affected by liver function. Although participants with known liver disease were excluded when possible, residual liver dysfunction may not have been entirely ruled out. Seventh, the dataset did not include information on histological subtypes of lung cancer, which limits our ability to interpret the observed sex-specific associations in more detail. To partially address this, we conducted stratified analyses by smoking status to account for etiological differences in lung cancer development. Eighth, in women, the number of smokers was too small to conduct meaningful stratified analyses by smoking status. While we performed stratified analyses by alcohol consumption status (e.g., never drinkers, current drinkers), interaction analyses by alcohol intake level could not be conducted due to insufficient sample size. Ninth, no participants withdrew consent during the follow-up period. Given the low emigration rate in Korea, attrition due to international relocation is expected to be minimal, although it cannot be entirely excluded.

## Figures and Tables

**Table 1:** Baseline characteristics of Korean Cancer Prevention Study-II participants *.

Characteristics	Men (n = 83,371)	Women (n = 50,225)	*p*-Value
Age, years	41.6 (9.5)	39.7 (10.7)	<0.0001
Body mass index ^†^	24.4 (2.9)	22.0 (3.0)	<0.0001
Total bilirubin, mg/dL	0.95 (0.38)	0.75 (0.30)	<0.0001
Direct bilirubin, mg/dL	0.36 (0.14)	0.29 (0.12)	<0.0001
Albumin, g/dL	4.58 (0.25)	4.45 (0.25)	<0.0001
Platelets, 10^3^/µL	246.27 (52.72)	257.82 (57.81)	<0.0001
Alcohol drinking, g/dL	22.67 (29.45)	5.98 (13.77)	<0.0001
Smoking status, %			
Never	22.7	89.4	<0.0001
Previous	32.7	6.4	<0.0001
Current	44.6	4.2	<0.0001
Any alcohol use, %			
Never	5.8	30.8	<0.0001
Previous	7.9	16.5	<0.0001
Current	86.3	52.7	<0.0001

* Data are expressed as mean (SD) unless otherwise indicated. Participants with any of the following features at study entry were excluded: missing data on serum bilirubin level, existing cancer, and missing data on smoking status. ^†^ Body mass index was calculated as weight in kilograms divided by the square of height in meters.

**Table 2:** Quartile and trend analysis of the association between total bilirubin, ALBI, and PALBI and lung cancer by sex.

	Indicators	Case (Median Follow-Up Time, IQR)	Q1HR (95% CI) §	Q2HR (95% CI) §	Q3HR (95% CI) §	Q4HR (95% CI) §	*p*-Value for Trend
Men	Total Bilirubin	553 (14.0, 13.4–14.6)	1	0.98 (0.78–1.22)	0.79 (0.62–1.00)	0.71 (0.56–0.91)	0.0014
	ALBI	553 (14.0, 13.4–14.6)	1	1.00 (0.79–1.26)	0.78 (0.61–0.99)	0.73 (0.57–0.93)	0.0021
	PALBI	523 (14.0, 13.4–14.6)	1	0.87 (0.68–1.12)	1.38 (1.09–1.74)	1.40 (1.10–1.79)	0.0003
Women	Total Bilirubin	168 (14.0, 13.4–14.6)	1	1.40 (0.97–2.03)	1.27 (0.81–2.01)	1.63 (1.03–2.58)	0.0420
	ALBI	168 (14.0, 13.4–14.6)	1	1.52 (1.03–2.22)	1.40 (0.91–2.15)	1.63 (1.01–2.63)	0.0433
	PALBI	153 (14.0, 13.4–14.6)	1	0.98 (0.56–1.60)	0.67 (0.41–1.11)	0.60 (0.37–0.97)	0.0082

Abbreviations: CI, confidence interval; HR, hazard ratio. § The Cox proportional hazards model was adjusted for age, sex, body mass index, smoking status, alcohol use, GOT, and GGT.

**Table 3:** Associations of a 1SD increase in total bilirubin, ALBI, and PALBI with lung cancer risk according to smoking status in men.

Indicators	Smoking Status	Case	HR (95% CI) §	*p*-Value
Total Bilirubin	Never-Smokers	57	0.85 (0.64–1.12)	0.2465
	Former Smokers	196	0.83 (0.71–0.97)	0.0177
	Current Smokers	300	0.85 (0.74–0.97)	0.0154
	Ever-Smokers *	496	0.79 (0.71–0.87)	<0.0001
ALBI	Never-Smokers	57	0.85 (0.65–1.12)	0.2515
	Former Smokers	196	0.84 (0.72–0.97)	0.0205
	Current Smokers	300	0.90 (0.80–1.01)	0.0749
	Ever-Smokers *	496	0.82 (0.75–0.90)	<0.0001
PALBI	Never-Smokers	57	1.04 (0.81–1.35)	0.7480
	Former Smokers	179	1.23 (1.05–1.44)	0.0091
	Current Smokers	287	1.14 (1.00–1.29)	0.0494
	Ever-Smokers *	466	1.26 (1.14–1.38)	<0.0001

Abbreviations: CI, confidence interval; HR, hazard ratio. * Ever-smokers include both current and former smokers. § The Cox proportional hazards model was adjusted for age, body mass index, alcohol use, GOT, and GGT.

**Table 4:** Associations of a 1SD increase in total bilirubin, ALBI, and PALBI with lung cancer risk according to alcohol consumption status in men.

Indicators	Alcohol Consumption Status	Case	HR (95% CI) §	*p*-Value
Total Bilirubin	Never-Drinkers	63	0.90 (0.66–1.21)	0.4742
	Former Drinkers	48	0.76 (0.53–1.07)	0.1191
	Current Drinkers	442	0.83 (0.75–0.92)	0.0006
	Ever-Drinkers *	490	0.82 (0.74–0.91)	0.0002
ALBI	Never-Drinkers	63	1.00 (0.78–1.29)	0.9933
	Former Drinkers	48	0.78 (0.58–1.06)	0.1129
	Current Drinkers	442	0.85 (0.77–0.94)	0.0015
	Ever-Drinkers *	490	0.84 (0.77–0.93)	0.0004
PALBI	Never-Drinkers	60	0.93 (0.71–1.23)	0.6203
	Former Drinkers	47	1.20 (0.88–1.65)	0.2534
	Current Drinkers	416	1.20 (1.09–1.33)	0.0004
	Ever-Drinkers *	463	1.20 (1.09–1.33)	0.0002

Abbreviations: CI, confidence interval; HR, hazard ratio. * Ever-drinkers include both current and former drinkers. § The Cox proportional hazards model was adjusted for age, smoking status, body mass index, GOT, and GGT.

**Table 5:** Associations of a 1SD increase in total bilirubin, ALBI, and PALBI with lung cancer risk according to alcohol consumption status in women.

Indicators	Alcohol Consumption Status	Case	HR (95% CI) §	*p*-Value
Total Bilirubin	Never-Drinkers	84	1.25 (1.00–1.56)	0.0548
	Former Drinkers	22	1.23 (0.76–1.98)	0.3949
	Current Drinkers	62	1.09 (0.82–1.45)	0.5541
	Ever-Drinkers *	84	1.12 (0.87–1.43)	0.3798
ALBI	Never-Drinkers	84	1.23 (0.98–1.54)	0.0798
	Former Drinkers	22	1.19 (0.76–1.86)	0.4393
	Current Drinkers	62	1.16 (0.89–1.50)	0.2800
	Ever-Drinkers *	84	1.16 (0.93–1.45)	0.1978
PALBI	Never-Drinkers	74	0.83 (0.64–1.06)	0.1326
	Former Drinkers	21	0.73 (0.47–1.15)	0.1761
	Current Drinkers	58	0.83 (0.63–1.09)	0.1782
	Ever-Drinkers *	79	0.81 (0.64–1.02)	0.0757

Abbreviations: CI, confidence interval; HR, hazard ratio. * Ever-drinkers include both current and former drinkers. § The Cox proportional hazards model was adjusted for age, smoking status, body mass index, GOT, and GGT.

## Data Availability

The summary statistics generated during and/or analyzed during the current study are available from the corresponding author on reasonable request. The R code generated during and/or analyzed during the current study is available from the corresponding author on reasonable request.
